# Astragaloside IV Protects Detrusor from Partial Bladder Outlet Obstruction-Induced Oxidative Stress by Activating Mitophagy through AMPK-ULK1 Pathway

**DOI:** 10.1155/2022/5757367

**Published:** 2022-07-13

**Authors:** Xiaoyu Zhu, Fangze Tao, Li Zhang, Yajie Yu, Yanchao Xu, Gang Yang, Zhifeng Wei, Yidong Cheng, Xuelai Yin, Xinyuan Zhang, Wu Wei, Anxi Wang

**Affiliations:** ^1^Department of Urology Surgery, Nanjing Hospital of Chinese Medicine Affiliated to Nanjing University of Chinese Medicine, Nanjing 210000, China; ^2^Department of Urology Surgery, Jiangsu Provincial Hospital of Chinese Medicine Affiliated to Nanjing University of Chinese Medicine, Nanjing 210000, China; ^3^School of Chinese Traditional Surgery, Nanjing University of Chinese Medicine, Nanjing 210023, China

## Abstract

**Aims:**

Bladder outlet obstruction (BOO) and the consequent low contractility of detrusor are the leading causes of voiding dysfunction. In this study, we aimed to evaluate the pharmacological activity of astragaloside IV (AS-IV), an antioxidant biomolecule that possess beneficial effect in many organs, on detrusor contractility and bladder wall remodeling process.

**Methods:**

Partial BOO (pBOO) was created by urethral occlusion in female rats, followed by oral gavage of different dose of AS-IV or vehicle. Cystometric evaluation and contractility test were performed. Bladder wall sections were used in morphology staining, and bladder tissue lysate was used for ELISA assay. Primary smooth muscle cells (SMCs) derived from detrusor were used for mechanism studies.

**Results:**

Seven weeks after pBOO, the bladder compensatory enlarged, and the contractility in response to electrical or chemical stimuli was reduced, while AS-IV treatment reversed this effect dose-dependently. AS-IV also showed beneficial effect on reversing the bladder wall remodeling process, as well as reducing ROS level. In mechanism study, AS-IV activated mitophagy and alleviated oxidative stress via an AMPK-dependent pathway.

**Conclusion:**

Out data suggested that AS-IV enhanced the contractility of detrusor and protected the bladder from obstruction induced damage, via enhancing the mitophagy and restoring mitochondria function trough an AMPK-dependent way.

## 1. Introduction

Voiding dysfunction is a common health problem in the general population. As a vital component of the excretory system, the smooth muscle layer of the bladder wall (the detrusor smooth muscle) endows it with contractile function. With the extension of average life, the prevalence of underactive detrusor function, which stems from numerous etiologies such as benign prostatic hyperplasia (BPH) [1], is reported to be as high as 48% in adults [2]. The increased prostate volume compresses the urethra and leads to bladder outlet obstruction (BOO). Clinical assay suggests that the severity of BOO is highly associated with the oxidative stress level in the bladder wall [3]. Oxidative stress reportedly blocks the muscarinic receptor and reduces the detrusor contractility [4], as well as induces protein damage and activates apoptotic pathways [5], hence results in deterioration in bladder function. Discovering the pharmacological effect of antioxidant compound on detrusor may present protective strategies that enhances bladder contractility and alleviates voiding dysfunction.

Traditional Chinese medicine is important druggery storeroom and one of the main sources for the discovery of new lead compounds. Astragali Radix (AR, dried root of *Astragalus membranaceus*) have a long history of medicinal use in traditional Chinese medicine, and recent studies suggest that it possesses beneficial antioxidant effects [6]. Astragaloside IV (AS-IV), the major and main active substances of AR, reportedly protects the vascular smooth muscle cells from mitochondrial dysfunction and oxidative stress [7]. Considering the antioxidant benefits of AS-IV [8–10] as well as the resemblances between detrusor smooth muscle cells (SMCs) and vascular SMCs, whether AS-IV also possess a beneficial effect on BOO-induced detrusor dysfunction is worth to study.

In this research, we discovered the effect of AS-IV on detrusor function in partial BOO (pBOO) rat, a rodent model that recapitulates the salient features of detrusor oxidative stress, apoptosis, and bladder fibrosis in humans with urinary tract obstruction [11]. Our results suggested that AS-IV activates contractility as well as alleviates the oxidative stress of detrusor, via activating mitophagy through an AMPK-ULK1-dependent way.

## 2. Materials and Methods

### 2.1. Chemical Reagents

High purity AS-IV (≥98%, #A111275) and sodium carboxymethyl cellulose (CMC-Na) were purchased from the Aladdin Company. Compound C was purchased from the MedChemExpress (#HY-13418A). For animal studies, 1% CMC-Na was used as solubilizer to prepare 4% and 2% (g : ml) AS-IV suspension. 25 or 50 mg/kg body weight of AS-IV was garaged daily according to the experiment schedule. The control group and pBOO group were gavage by equal volume of 1% CMC-Na. For *in vitro* studies, AS-IV was prepared as a 10 mM stock in dimethyl sulfoxide (DMSO) and used at the concentration of 20 *μ*M or 40 *μ*M to treat cells. For blocking AMPK pathway, 10 *μ*M compound C was added to cell culture for an indicated time.

### 2.2. Creation of pBOO Model

Female Sprague-Dawley rats (200-250 g), purchased from the Gempharmatech (Nanjing, China), had access to food and water ad libitum and were subjected to 12 hour light/dark cycles; they were anesthetized with isoflurane and underwent microsurgical creation of pBOO essentially as described [12]. Briefly, anesthetized rats underwent a lower abdominal incision to expose the bladder. A 22 g polypropylene catheter was placed adjacent to the bladder outlet, and a sterile surgical ligature was placed snuggly around the bladder outlet and catheter. The catheter was then removed, and the abdominal incision was closed with sterile silk (muscular layer) and sterile surgical clamps (skin). Another group of rats received a sham-operation that consisted of surgically exposing the lower rat abdomen and briefly compressing the bladder outlet with sterile forceps.

All animal studies were performed according to guidelines established by the Research Animal Care Committee of Nanjing Medical University, China (Permit Number: IACUC-NJMU 1404075).

### 2.3. Cystometric Evaluation

Rats were anesthetized with urethane (1.5 g/kg). The bladder were exposed via a midline abdominal incision followed by removal of pubic symphysis and the medial portion of the pubic bones. A three-way valve-connected catheter (Becton, Dickinson and Company, Franklin Lakes, NJ, United States) was inserted into the top of bladder, the other two ends of the pipeline were connected to a peristaltic pump (Chonry Co., Nanjing, China) and a pressure transducer (Chengyi Co., Chengdu, China). Cystometric measurement was performed by infusing warm saline at a rate of 0.2 ml/min. The transduced pressure were recorded by a signal processing system (RM6240C; Chengyi Co.).

### 2.4. Tissue Harvest

After 4 weeks gavage, the rats were euthanized, and bladders for contractility testing were immediately immersed in ice-cold Krebs' solution (120 mM NaCl, 5.9 mM KCl, 25 mM NaHCO_3_, 1.2 mM Na_2_H_2_PO_4_, 1.2 mM MgCl_2_. 6H_2_O, 2.5 mM CaCl_2_, and 11.5 mM dextrose) with minimal handling. Tissues used for molecular analysis were immediately snap-frozen in liquid nitrogen, while tissues used for morphological staining were fixed with 4% paraformaldehyde.

### 2.5. Contractility Testing

Functional analysis of muscle strips was performed as described previously [13]. Briefly, bladder SM strips were prepared by removing the mucosa under stereomicroscope vision. Bladder tissue was attached to a force transducer (Grass Technologies), suspended in Krebs solution in an organ bath, and maintained at 37°C to equilibrate for 1 hour bubbled with a mixture of 95% O2 and 5% CO_2_ under a force of 0.5 grams. Contractile responses to carbachol (1 nM to 10 *μ*M), 120 mM KCl, 10 *μ*M *α*, *β*-methyl-ATP, and to EFS (1 to 64 Hz, 40 V, 0.5 ms pulse width, 10-second duration) were measured. Data were calculated as force (millinewtons) normalized by tissue cross-sectional area and are expressed as means ± SEM.

### 2.6. Primary Detrusor Cell Separation

Primary detrusor smooth muscle cells from each model group were separated as previously described [14]. Briefly, isolated bladder tissue from the sacrificed rats were cut to small pieces, the muscle layer were cleared from the mucosa, epithelium, and other attached tissue under a dissection microscope. Separated muscle tissue were cut into fine sand like particles and digested with 10 U/ml elastase (type III), 150 U/ml collagenase (type I), and 2.0 mg/ml bovine serum albumin (all purchased from Sigma). After incubation at 37°C for 90 min (mix every 5 min), the cells were filtered through a 100 *μ*M cell strainer and centrifuged at 3000 rpm for 5 min. The obtained smooth muscle cells were resuspended in DMEM medium supplemented with 10% fetal bovine serum (Hyclone) and seeded into indicated dish. Once the cells adhere to the wall, they were harvested, measured, or immune-stained.

### 2.7. Immunoblot

For isolation of protein in bladder tissue, a commercial kit was used following its protocol (Beyotime). The primary antibodies were as follows (dilution 1 : 1000): rabbit anti-Pink1 (#23274-1-AP, Proteintech), rabbit anti-Prkn (#14060-1-AP, Proteintech), rabbit anti-LC3 (#14600-1-AP, Proteintech), rabbit anti- P62 (#18420-1-AP, Proteintech), mouse anti-Phospho-AMPK*α* (Thr12) (#50081, Cell Signaling), rabbit anti-AMPK*α* (#2532, Cell Signaling), rabbit anti-Phospho-Ulk1(Ser555) (#5869, Cell Signaling), and rabbit anti-Ulk1 (#8054, Cell Signaling). Secondary antibodies were anti-mouse IgG and anti-rabbit IgG (Jackson Laboratory, West Grove, PA, USA). All blots were repeated three times using tissue lysates merged from six rats' samples in each group.

### 2.8. Immunostaining

Classic H&E and TUNEL staining were performed by the Servicebio Company (Wuhan, China). For immunohistochemistry staining, the bladder sections were immersed in citrate buffer (0.01 M, pH = 6.0) at 95°C for 5 min to perform heat-induced antigen retrieval. The primary antibodies used were rabbit anti-*α*SMA (#19245, Cell Signaling), rabbit anti-Col I (#72026, Cell Signaling), mouse anti-Col III (#sc-271249, Santa Cruz), mouse anti mAChR M2 (#sc-33712, Santa Cruz), and mouse anti mAChR M3 (#sc-518107, Santa Cruz). DAB kits (Gene Tech, Shanghai, China) were then used to show the immunohistochemical staining, and the nuclei were stained with haematoxylin. Images were obtained with a DP70 camera (Olympus, Tokyo, Japan).

### 2.9. Fluorescence and Confocal Imaging

Primary detrusor cells were plated on glass bottom imaging dishes, and stained with each dyes as indicated. Nuclei were marked with 500 nM Hoechest 33342; lysosomes were labeled with LysoTracker Green (100 nM); mitochondria were marked with 100 nM Tomm20 Red, according to the manufacturer's instructions. Cells were imaged using Zeiss LSM710 confocal microscope.

### 2.10. Mitochondrial Respiratory Function Assay

A Seahorse XF96e Analyzer (Seahorse Bioscience—Agilent, Santa Clara, CA, USA) was used to measure the mitochondrial respiratory function. In this study, 5∗10^4^ primary detrusor cells were plated in each well, followed by indicated treatment. Before the assay, the growth medium were replaced with the Seahorse XF assay media (Eagle's modified Dulbecco's medium, w/o glucose, pH = 7.4; Agilent Seahorse) to which 1 mM pyruvate, 10 mM glucose, and 2 mM L-glutamine were supplemented. Cells was incubated without CO_2_ for 1 h and followed by measuring the baseline and stimulated oxygen consumption rate (OCR) (pmol/min). Three reagents were used while the sequential OCR measurements were performed: 1.0 *μ*M oligomycin (an ATP synthase inhibitor of the electron transport system respiratory chain complex V), 1.5 *μ*M carbonyl cyanide 4-(trifluoromethoxy) phenylhydrazone (FCCP) (uncoupler), and 0.5 *μ*M rotenone with antimycin A (inhibitor of electron transport system respiratory complex I/III).

### 2.11. Measure of Inflammatory Cytokines

For the detection of bladder tissue inflammatory cytokines, 200 *μ*g bladder tissue were dissected, and the whole layer tissue was homogenized in ice-cold normal saline and followed by centrifuged at 1,500 ×g for 15 min at 4°C. The supernatant were analyzed by each corresponding kit, IL-1*β*: #H002; IL-6: #H007-1-1; IL-10: #H024; and TNF*α*: #H052-1. All four kits were purchased from the Nanjing Jiancheng Bioengineering Institute.

### 2.12. Measure of Oxidative Stress

For testing the accumulation of reactive oxygen species (ROS), malondialdehyde (MDA), the activities of glutathione peroxidase (GSH), and superoxide dismutase (SOD), primary detrusor cells were separated and homogenized in ice-cold normal saline and followed by centrifuged at 1,500 ×g for 15 min at 4°C. The supernatant were analyzed by each corresponding kit, ROS: #E004-1-1; SOD: #A001-3-2; GSH: #A005-1-2; and MDA: #A005-1-2. All four kits were purchased from the Nanjing Jiancheng Bioengineering Institute.

### 2.13. Real-Time PCR

Total RNA was extracted from bladder tissue with TRIzol (Invitrogen, #12183555), followed by precipitation with isopropanol and ethanol. Complimentary DNA (cDNA) was obtained by reverse transcription of 0.5 mg RNA using reverse transcription and cDNA synthesis kit (Takara, # 639506). Quantitative real-time PCR analysis was performed with SYBR Green Master Mix (Vazyme, Q111-02) and analyzed using a Roche Real-Time PCR System. Expression levels were determined using the relative standard curve method and normalized to the housekeeping gene Gapdh. Primer sequences for qPCR were list below. Gapdh: forward, TTGTCATGGGAGTGAACGAGA, reverse, CAGGCAGTTGGTGGTACAGG. Drp1: forward, CAGGAATTGTTACGGTTCCCTAA, reverse, CCTGAATTAACTTGTCCCGTGA. Mfn1: forward, CCTACTGCTCCTTCTAACCCA, reverse, AGGGACGCCAATCCTGTGA. Mfn2: forward, AGAACTGGACCCGGTTACCA, reverse, CACTTCGCTGATACCCCTGA. Opa1: forward, TGGAAAATGGTTCGAGAGTCAG, reverse, CATTCCGTCTCTAGGTTAAAGCG. Pink1: forward, TTCTTCCGCCAGTCGGTAG, reverse, CTGCTTCTCCTCGATCAGCC. Prkn: forward, GAGGTCGATTCTGACACCAGC, reverse, CCGGCAAAAATCACACGCAG.

### 2.14. Statistical Analysis

*In vivo* assay was repeated two times, with the number of mice included in each group indicated. Significant differences were assessed by using one-way ANOVA followed by the Student-Newman-Keuls (SNK) test. *P* < 0.05 was considered statistically significant.

## 3. Result

### 3.1. Experiment Design and General Information

**Figure 1:**
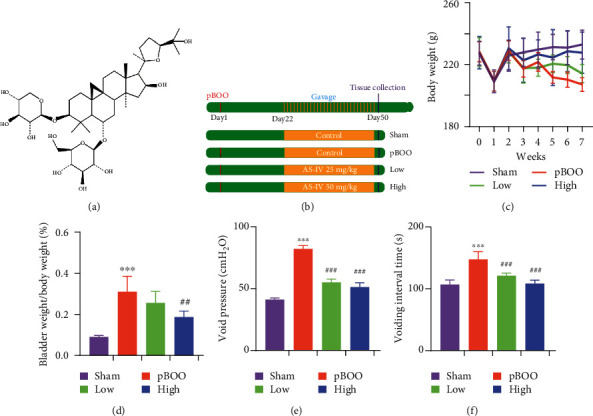
Overview of experiment schedule. (a) Chemical structure of astragaloside IV. (b) Experiment schedule and grouping information. (c) Body weight change during the experiment period. (d) Ratio of bladder weight/body weight. (e, f) Cystometric evaluation of voiding pressure (e) and voiding interval time (f). ^∗∗∗^*P* < 0.001, pBOO vs. sham; ^##^*P* < 0.01, ^###^*P* < 0.001, AS-IV-treated groups vs. pBOO. Error bars show ±SEM.

The pharmacological effect of purified AS-IV (Figure 1(a)) on pBOO-induced detrusor dysfunction was analyzed following experiment schedule (Figure 1(b)). Rats either subjected to pBOO (*n* = 6) or not (sham, *n* = 6) get a 3-week recovery period, then followed by gavages of control/AS-IV (25 mg/kg, low, *n* = 6)/AS-IV (50 mg/kg, high, *n* = 6) for another 4 weeks. Weekly measurement of the body weight showed a decreasing body mass in the pBOO group from the 5th week to the end point, while AS-IV treatment reversed this declination (Figure 1(c)). Increased urination pressure of pBOO model leads to bladder compensatory hypertrophy, as reflected by the raised ratio of bladder weight/body weight, AS-IV exposure dose-dependently reduced this bladder enlargement (Figure 1(d)). Cystometric measures including voiding pressure (Figure 1(e)) and voiding frequency (Figure 1(f)) differed between the pBOO and control groups, while AS-IV also showed beneficial effect.

### 3.2. AS-IV Treatment Ameliorates Bladder Wall Remodeling in pBOO Rats

**Figure 2:**
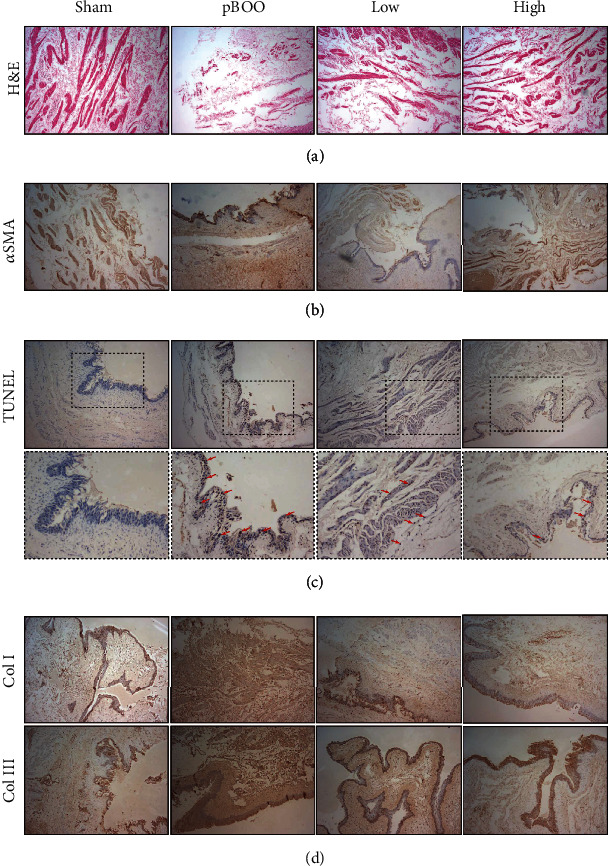
AS-IV treatment ameliorates bladder wall remodeling in pBOO rats. (a–d) Bladder wall sections of four groups were analyzed by morphology staining as indicated in panel. General structure (H&E) (a), muscle tissue (*α*SMA) (b), apoptosis (Tunel, black arrow pointed to parts of positive nuclear) (c), and fibrosis (Col I and Col III) (d) staining indicated that pBOO induced bladder wall remodeling, and AS-IV treatment alleviated this progress.

To further determine the effect of AS-IV on pBOO-induced bladder wall remodeling, we take histological and morphometric analysis by H&E, *α*SMA, Tunel, and Col I/III. In the group of pBOO, the bladder wall presented significant reduced muscle mass on H&E, compared to shams, while AS-IV treatment reversed this effect (Figure 2(a)). Compared with the precise staining in sham/low/high group, the *α*SMA staining in pBOO group was dispersed and blurry, which indicated the apoptosis of muscle cells and the leakage of antigens into the bladder tissue (Figure 2(b)). TUNEL assay showed clustered positive staining in epithelium and detrusor in pBOO model group, while after AS-IV exposure, apoptosis with in the smooth muscle is reduced (Figure 2(c)). Col I and Col III staining indicated high levels of fibrotic lesions in the bladders from pBOO group compared to sham rats, and AS-IV also reduced this effect dose-dependently (Figure 2(d)).

### 3.3. AS-IV Treatment Enhances the Contractile Force of Detrusor in pBOO Rats

**Figure 3:**
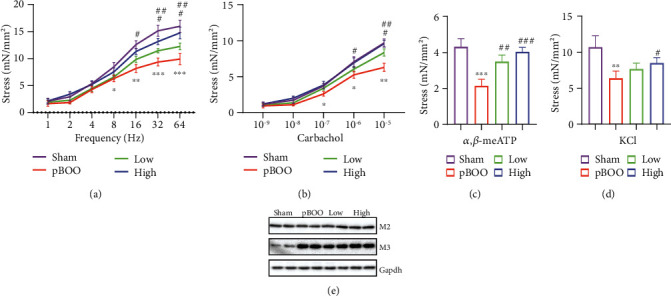
AS-IV treatment enhances the contractile force of detrusor in pBOO rats. (a–d) Contractile responses of bladder muscle strips from rats subjected to sham (violet), pBOO (red) followed by either control or low-dose AS-IV (greed)/high-dose AS-IV (blue) were determined by isometric tension testing in response to electrical field stimulation (a), carbachol (b), *α*, *β*-methyl-adenosine triphosphate (*α*, *β*-me ATP) (c), and potassium chloride (KCl) (d). (e) Protein level of mAChR M2 and M3 in bladder lysis. ^∗^*P* < 0.05, ^∗∗^*P* < 0.01, ^∗∗∗^*P* < 0.001, pBOO vs. sham; ^#^*P* < 0.05, ^##^*P* < 0.01, ^###^*P* < 0.001, AS-IV-treated groups vs. pBOO. Error bars show ±SEM.

Pharmacological evaluation of AS-IV on contractile activity of bladder tissue strips were then analyzed. Electrical stimulation (Figure 3(a)), cholinergic drug (carbachol) (Figure 3(b)), ATP (Figure 3(c)), and potassium channel activator (KCl) (Figure 3(d)) were used to induce contraction. Detrusors from obstructed mice displayed decreased contractility with all agonists compared with those from shams, while AS-IV treatment reversed this effect dose-dependently (Figures 3(a)–3(d)). The protein levels of M2 and M3 receptors were then analyzed, using bladder tissue lysis that contains the whole layer of bladder wall. The M3 level was upregulated in BOO group, while M2 was not changed (Figure 3(e)). Both muscarinic receptors were not affected by AS-IV treatment (Figure 3(e)).

### 3.4. AS-IV Treatment Reduces the Inflammation and Oxidative Stress in pBOO Rats

**Figure 4:**
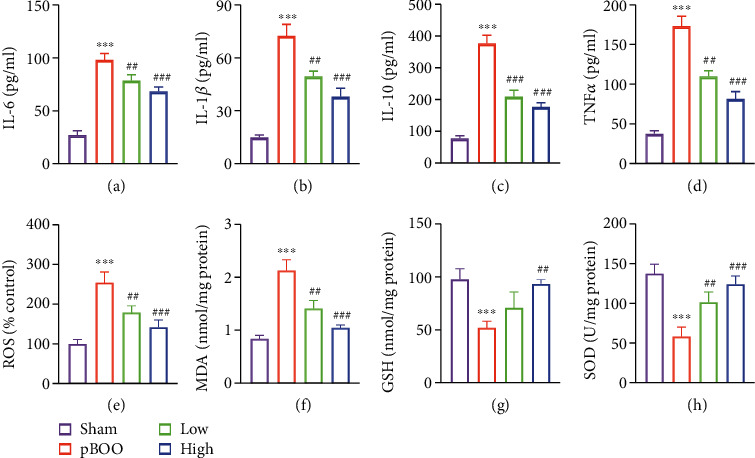
AS-IV treatment reduces the inflammation and oxidative stress in pBOO rats. (a–d) Inflammatory cytokines IL-1*β* (a), IL-6 (b), IL-10 (c), and TNF*α* (d) were determined by ELISA using bladder wall tissue lysate. (e–h) Oxidative stress levels were analyzed using detrusor SMCs of bladder wall. (e) ROS; (f) MDA; (g) GSH; and (h) SOD. Error bars show ±SEM. ^∗∗∗^*P* < 0.001, pBOO vs. sham; ^##^*P* < 0.01, ^###^*P* < 0.001, AS-IV-treated groups vs. pBOO. Error bars show ±SEM.

Using the bladder tissue lysate, the inflammatory cytokines IL-6 (Figure 4(a)), IL-1*β* (Figure 4(b)), IL-10 (Figure 4(c)), and TNF*α* (Figure 4(d)) were determined by ELISA. Consistent with morphology results, the cytokine levels upregulated in pBOO model group, and AS-IV reversed these inflammation responses (Figures 4(a)–4(d)).

AS-IV reportedly reduces oxidative stress [8–10], which plays central role in pBOO-induced detrusor underactivity and damage [4]. We then analyzed the oxidative stress level in primary detrusor cells separated from the bladder wall of the four groups. Compared with the sham group, the levels of cellular ROS (Figure 4(e)) and MDA (Figure 4(f)) were markedly increased, while the activity of antioxidative GSH (Figure 4(g)) and SOD (Figure 4(h)) were reduced in pBOO group. AS-IV dose-dependently reversed the aggravated oxidative damage induced by pBOO (Figures 4(e)–4(h)).

### 3.5. AS-IV Treatment Recovers Mitochondria Function and Activates Mitophagy in Primary Detrusor Cells

**Figure 5:**
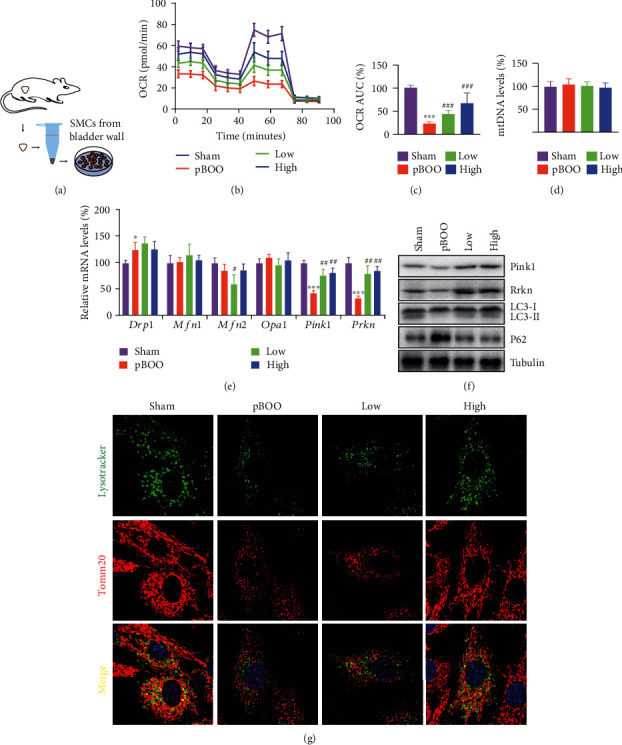
AS-IV treatment recovers mitochondria function and activates mitophagy in primary detrusor cells. (a) Experiment schedule: SMCs were separated from detrusor of each model group via an enzyme-based method. Once the cells attached to the wall, they were used for the studies below. (b, c) Analysis of mitochondrial respiration by OCR analysis. (d) Analysis of mitochondrial DNA levels. (e) Expression profile of marker genes regulating mitochondria fission, fusion, and mitophagy, using qPCR assay. (f) Western blot testing the protein level of Pink1, Prkn, Lc3, and P62. (g) Fluorescent staining of mitochondria (Tomm20, red) and lysosome (Lyso-Tracker, green). Error bars show ±SEM. ^∗^*P* < 0.05, ^∗∗∗^*P* < 0.001, pBOO vs. sham; ^#^*P* < 0.05, ^##^*P* < 0.01, ^###^*P* < 0.001, AS-IV-treated groups vs. pBOO. Error bars show ±SEM.

Oxidation of metabolic intermediates of the electron transport chain within mitochondria generates superoxide, and uncontrolled release of ROS will further damage mitochondria function [15]. We then analyzed the mitochondria function in primary detrusor cells separated from model rats (Figure 5(a)). Results of the sea-horse assay showed that AS-IV treatment raised both the baseline and maximal oxygen consumption rate (OCR) in smooth muscle cells of pBOO rats (Figures 5(b) and 5(c)). However, the mitochondria content were not affected by either pBOO operation or by AS-IV treatment (Figure 5(d)).

Mitochondria are highly dynamic organelles, which biology function is delicately modulated through fission, fusion, and mitophagy [16]. Through qPCR assay, the mRNA level of key regulators that govern mitochondria dynamic are profiled. Mitochondria fission, as reflected by the dynamin-related protein 1 (Drp1), and mitochondria fusion, as reflected by the mitofusin1 (Mfn1), Mfn2, and optic atrophy 1 (OPA1), were not affected after AS-IV exposure, while the expression level of mitophagy gene Pten-induced putative kinase 1 (Pink1) and Parkin (Prkn) were significantly decreased in pBOO group and upregulated after AS-IV treatment (Figure 5(e)). Protein level of Pink1, Prkn, and autophagic flux, as represented by LC3II and P62, showed same trend after AS-IV treatment (Figure 5(f)). The florescent staining results revealed that compared to the cells from pBOO rats, AS-IV treatment significantly increased the costaining of mitochondrial and lysosome, which represent mitophagy (Figure 5(g)).

### 3.6. AS-IV Activates Mitophagy through an AMPK-Dependent Way

**Figure 6:**
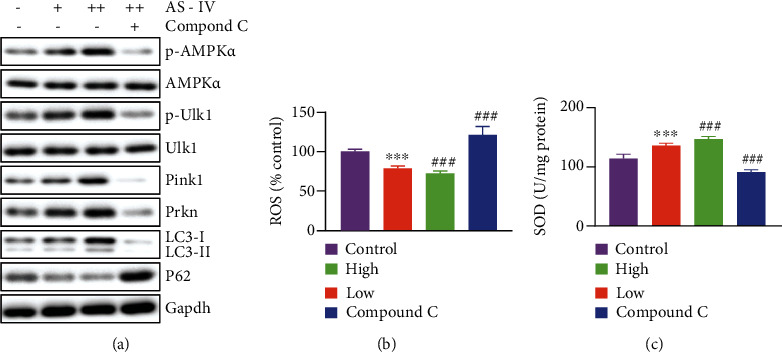
AS-IV activates mitophagy through an AMPK-dependent way. (a–c) Detrusor SMCs were separated from the bladder of healthy rats and seeded into 12-well plate. Cells were treated with 20 *μ*M (+/low) or 40 *μ*M (++/high) tangeretin, with or without 10 *μ*M compound C, for 24 h. (a) The protein levels of phospho-/total-AMPK and Ulk1, as well as mitophagy marker genes were determined by immunoblotting. (b, c) Oxidative stress levels were analyzed, as reflected by ROS level (b) and SOD activity (c). Error bars show ±SEM. ^∗∗∗^*P* < 0.001, low vs. control; ^###^*P* < 0.001, AS-IV-treated groups vs. pBOO. Error bars show ±SEM.

AS-IV reportedly enhances AMPK activity [17], a master regulator of autophagy/mitophagy. Once activated, AMPK phosphorylates unc-51-like kinase 1 (Ulk1) and starts a signal pathway that initiates autophagosome formation [18]. Using separated detrusor cells from normal rats, the underlying mechanisms of AS-IV on activating mitophagy were then examined. Western blots results suggested that protein level of Pink1, Prkn, and autophagic flux were upregulated after AS-IV treatment, while blocking AMPK with its inhibitor compound C completely reversed these effects (Figure 6(a)). Measurement of the cellular ROS accumulation (Figure 6(b)) and SOD activity (Figure 6(c)) revealed an antioxidant activity after AS-IV exposure, and blocking AMPK pathway completely reversed these effects (Figures 6(b) and 6(c)).

## 4. Discussion

In the present study, we determined the protect effect of AS-IV on pBOO rats-an experimental model for detrusor dysfunction and bladder wall remodeling. Currently, no effective pharmacotherapy exists to restore detrusor contractility in voiding dysfunction patients. Although pharmacological treatments, such as alpha-blockers [19], for underactive bladder -(UAB-) related BOO were effective, they showed no effect on detrusor contractility [20]. Parasympathomimetics can theoretically enhance detrusor contractility because they augment excitatory acetylcholine action between the synapses. However, muscarinic agonists or cholinesterase inhibitors have shown limited efficacy and adverse effects on restoration of detrusor contraction [21]. Hence, investigation of novel therapeutic strategies that is enhancing detrusor function is warranted. In our study, AS-IV enhanced the contractility of detrusor tissue, reduced the oxidative stress and activated mitophagy through an AMPK-ULK1-dependent way, and presented a promising pharmacological treatment.

Bladder wall remodeling, such as collagen deposition, fibrosis in submucosa, and bladder wall apoptosis, happens after pBOO. Theoretically, in conditions such as BPH or BOO, the bladder contracts against an obstructed outlet and went through a two-stage change. The initial response is adaptive, involving a compensatory phase of smooth muscle hypertrophy to overcome the increased outlet resistance. When the demand outstrips the adaptive capability, the bladder remodels and ultimately leading to a loss of detrusor contractility as the bladder decompensates [22]. In the well-studied rodent model, the contractile forces in response to electrical field stimulation, carbachol, and KCl in 4-weeks BOO rats were 30.7-51.7% of those in control rats [23]. Similar results were observed in several mouse-based BOO studies [24, 25]. In our study, tissue samples were collected in 7 weeks BOO model, a more severe decompensating stage with detrusor underactivity. Using multiple morphology staining and ELISA method, our data suggested that AS-IV dose-dependently reduced bladder tissue apoptosis, fibrosis, and inflammation, as well as increased the contractile capacity. Though AS-IV showed protective effect in BOO model at the time point of 7 weeks, short-term study are still needed to fully delineate the contribution of AS-IV to the development of BOO induced bladder dysfunction at different time points.

Many factors contribute to the low detrusor contractility following BOO, including impaired mitochondria function. During detrusor contraction, the obstructed bladder should increase adenosine monophosphate (AMP)/adenosine triphosphate (ATP) ratio [26], together with a reduced activity of mitochondria function [27]. The damaged mitochondria contribute produced energy and generates more peroxides; both of which weakens detrusor contractility. Interestingly, our data and previous study [28] showed that even though the AMP/ATP ratio increased in BOO animals, the activities of AMPK, energy sensor that governs the homeostasis status of the cell, were blocked. This inconsistency could partly be due to the reduced phosphorylation of AMPK upstream kinase, such as Ca2^+^/calmodulin-dependent protein kinase (CaMKK*β*) [28]. Further research into the acknowledged gaps in this area is needed in the future.

Direct measurements of such residual volumes were not performed in this study. We cannot rule out the possibility that a diverse postvoiding residual volume may contribute to the changed cystometric data. But consistent results were also observed in contractility testing using separated muscle strip in vitro, which was certainly not affected by the residual urine volume. Therefore, our data showed that AS IV enhanced the contractile capacity. However, the expression of muscarinic receptors in bladder wall were not changed after AS-IV treatment. It seems AS-IV enhanced contractility not by restoring the expression of muscarinic receptors, but by protecting the detrusor from oxidative stress and apoptosis. What is more, the AS-IV was systemic administrated by gavage in this study; its biofunction on the central nervous system cannot be ignored and should be studied in the future via patch clamp-based methods.

Through targeting multiple genes and downstream pathways such as AMPK [29], Hif-1*α* [30], and TLR4 [31], AS-IV demonstrated potential cardio-protective and immunological enhancement activities through experimentation *in vitro* and *in vivo* [32]. In this research, pBOO suppressed the AMPK-Ulk1 signaling, while AS-IV treatment reversed this effect. The AMPK-Ulk1 pathway plays central role in regulating mitophagy. The AMPK complex senses mitochondrial damages and activates Ser/Thr kinase ULK1, which represents the most upstream activator of the autophagic pathway [33]. Regarding mitophagy, ULK1 is phosphorylated at Ser555 by AMPK, which triggers ULK1 translocation to mitochondria and mitophagy [34, 35]. In our study, the phosphorylated AMPK/Ulk1 were downregulated in pBOO group, while AS-IV reversed this effect dose-dependently. Hence, the role of AS-IV in promoting mitophagy likely acts through the AMPK-Ulk1 pathway. Using separated primary detrusor SMCs, blocking AMPK reversed the antioxidant and mitophagy promoting effect of AS-IV; however, limited by the lack of *in vitro* model of pBOO, whether blocking the AMPK-Ulk1 pathway could reverse AS-IV's effect on detrusor contractility is still unknown and needs further study.

In conclusion, our results suggest that AS-IV, key ingredient of Astragali Radix, is a detrusor protector that restore muscle contractility and ameliorates bladder wall remodeling in pBOO model, via activating mitophagy and restoring mitochondria function through AMPK-Ulk1 pathway.

## Data Availability

The data that support the findings of this study are available from the corresponding authors upon reasonable request.
